# A Combinatorial Cell and Drug Delivery Strategy for Huntington’s Disease Using Pharmacologically Active Microcarriers and RNAi Neuronally-Committed Mesenchymal Stromal Cells

**DOI:** 10.3390/pharmaceutics11100526

**Published:** 2019-10-12

**Authors:** Emilie M. André, Gaëtan J. Delcroix, Saikrishna Kandalam, Laurence Sindji, Claudia N. Montero-Menei

**Affiliations:** 1 CRCINA, UMR 1232, INSERM, Université de Nantes, Université d’Angers, F-49933 Angers, France; emilie.andre01@gmail.com (E.M.A.); kandalamsai@gmail.com (S.K.); laurence.sindji@univ-angers.fr (L.S.); 2 Geriatric Research, Education, and Clinical Center, and Research Service, Bruce W. Carter Department of Veterans Affairs Medical Center, Miami, FL 33125, USA; gdelcroix@nova.edu; 3 College of Allopathic Medicine, Nova Southeastern University, Fort Lauderdale, FL 33125, USA

**Keywords:** tissue engineering, Huntington’s disease, siRNA, nanoparticles, microcarriers, mesenchymal stromal cells

## Abstract

For Huntington’s disease (HD) cell-based therapy, the transplanted cells are required to be committed to a neuronal cell lineage, survive and maintain this phenotype to ensure their safe transplantation in the brain. We first investigated the role of RE-1 silencing transcription factor (REST) inhibition using siRNA in the GABAergic differentiation of marrow-isolated adult multilineage inducible (MIAMI) cells, a subpopulation of MSCs. We further combined these cells to laminin-coated poly(lactic-*co*-glycolic acid) PLGA pharmacologically active microcarriers (PAMs) delivering BDNF in a controlled fashion to stimulate the survival and maintain the differentiation of the cells. The PAMs/cells complexes were then transplanted in an ex vivo model of HD. Using Sonic Hedgehog (SHH) and siREST, we obtained GABAergic progenitors/neuronal-like cells, which were able to secrete HGF, SDF1 VEGFa and BDNF, of importance for HD. GABA-like progenitors adhered to PAMs increased their mRNA expression of NGF/VEGFa as well as their secretion of PIGF-1, which can enhance reparative angiogenesis. In our ex vivo model of HD, they were successfully transplanted while attached to PAMs and were able to survive and maintain this GABAergic neuronal phenotype. Together, our results may pave the way for future research that could improve the success of cell-based therapy for HDs.

## 1. Introduction

Huntington’s disease (HD) is a genetic neurodegenerative disorder caused by the abnormal repetition of CAG nucleotides in the Huntingtin (HTT) gene. This leads to a pathological expansion of polyglutamine (polyQ) and aggregation of the mutated HTT protein in the brain, more specifically in the striatum [1,2]. HD is characterized by a progressive degeneration of striatal GABAergic medium spiny projection neurons, followed by a progressive degeneration extending throughout the brain [3]. Clinically, this results in involuntary movements, cognitive impairment and psychiatric manifestations, culminating in death around 15–20 years after the onset of motor symptoms [4,5]. Currently, there is no proven medical therapy to alleviate the onset or progression of HD [6].

Mesenchymal stromal cells (MSCs), have emerged for clinical transplantation studies due to their easy availability, their immune-modulatory properties [7] and their capacity to release neurotrophic factors and to create a neuroprotective microenvironment [8]. Clinical trials using MSCs in the central nervous system are now underway for many neurological disorders and have shown the feasibility of this approach [9]. Pre-clinical studies with HD models have shown improvement in behavior and a reduced lesion volume after MSC implantation. These beneficial effects could be explained by the secretion of neurotrophic factors including brain-derived neurotrophic factor (BDNF), ciliary neutrotrophic factor, nerve growth factor, insulin-like growth factor 1 and epidermal growth factor (EGF) [10,11]. The trans-differentiation of MSCs into a neural/neuronal lineage, even if possible in vitro, is far less efficient in vivo and their functional maturity remains too scarce [12]. The rationale for their transplantation does not lie on their capacity to replace the damaged neuronal cells, but on their ability to neuroprotect and repair by their paracrine effects on the surrounding environment [8]. Even though they are not used in a cell replacement strategy demanding electrophysiolocally functional connections, if the cells are to be safely used in clinical trials, it is important that they blend into the microenvironment of the brain and present the same neuronal phenotype. Therefore, MSCs are required to be committed to a neuronal cell lineage and maintain this phenotype to ensure their safe transplantation in the brain. In this regard, REST/NRSF a repressor transcription factor functioning as a master negative regulator of neurogenesis by binding to a specific DNA domain named RE1 motif is an interesting target to inhibit and thus induce a neuronal specification [13,14]. It was thus recently shown that the silencing of REST, obtained by a recombinant lentivirus carrying a small interfering RNA (siRNA) for REST, induced a neural/neuronal differentiation of MSCs [15,16].

Nanoparticles have been developed to efficiently and safely deliver siRNA both in vitro and in vivo and to avoid insertional gene mutations and viral toxicity issues. We recently designed lipid nanocapsules (LNC) able to encapsulate siRNAs complexed to lipids, thus protecting the siRNA from degradation. LNCs consisting of a lipid liquid core of triglycerides and a rigid shell of lecithin and polyethylene glycol (PEG) can be formulated by a simple and easily industrialized solvent-free process based on the phase inversion of an emulsion [17,18]. They have a high stability and can destabilize lysosome’s membranes by a proton sponge effect [19]. We also demonstrated that LNCs associated with siREST in MSCs were able to induce their neuronal commitment with a better efficiency than the commercial reagent Oligofectamine^®^ [9]. However, MSCs are a heterogeneous population presenting different differentiation properties. A homogeneous subpopulation of MSCs, termed marrow-isolated adult multilineage inducible (MIAMI) cells, which present a unique genetic profile expressing several pluripotency markers (Oct4, Sox2, Nanog, SSEA4), share many proteins with embryonic stem cells, secrete more tissue repair factors than MSCs, protect the neurovasculature and can be induced to differentiate into cells from all three germ layers [20,21,22], emerge as a good alternative for cell therapy studies. MIAMI cells can be specified into the neuronal lineage with epidermal growth factor (EGF) and fibroblast growth factor-2 (FGF-2) [23] and induced to an immature neuronal dopaminergic phenotype presenting appropriate electrophysiological properties, regardless of the donor age, with a three-step protocol in vitro requiring neurotrophins [24,25]. In addition, laminin (LM) was shown to enhance the neuronal differentiation of these cells [8]. However, further efforts are needed to maintain a differentiated phenotype and increase engraftment/survival after transplantation in the brain.

Pharmacologically active microcarriers (PAMs) are biodegradable and biocompatible poly(lactic-*co*-glycolic acid) PLGA-based microspheres covered with extracellular matrix molecules (ECM) such as fibronectin or laminin providing an adequate three-dimensional (3D) biomimetic surface for the transplanted cells and thus enhancing their survival [22,24]. Moreover, PAMs can also release in a controlled manner an encapsulated growth factor allowing a better cell engraftment by stimulating the transplanted cells and/or the microenvironment [22,26]. In this context, PAMs transporting human stem cells and delivering different growth factors have been shown to be beneficial in several animal models of neurological disorders, cartilage and cardiac pathologies [27,28,29,30]. In this regard, BDNF, which is involved in neuronal GABAergic differentiation and neuronal survival [30,31,32] may maintain the differentiated phenotype and increase the survival of the transported MIAMI cells. Moreover, in the case of HD, several studies demonstrated that the expression of BDNF is reduced in the patient’s brains [33]. Promising results show that BDNF supplementation increases the survival of enkephalin-immunoreactive striatal neurons, reduces striatal interneuronal loss and improves motor function in HD animal models [31,34,35]. PAMs would have the benefit to prevent BDNF degradation while providing for its controlled release over time, hence maximizing the possible benefits of BDNF for neuroprotection in HD. 

In this study, we aim to first improve the neuronal differentiation of a subpopulation of MSCs, the MIAMI cells, as well as to study their behavior after combining them with PAMs releasing BDNF in vitro and on organotypic brain cultures. We first evaluated the impact of siREST LNC transfection on the neuronal commitment of EGF/FGF-2 pre-treated MIAMI cells. We set up a differentiation protocol based mainly on sonic hedgehog (SHH) treatment followed by BDNF as previously described [36,37], to evaluate the GABAergic differentiation potential of these cells in vitro; GABAergic cells being the predominant cell type present in the striatum. We thus also studied the effects of the REST nanocapsule-silencing on MIAMI cells induction towards the GABAergic phenotype. The secretome and neuronal marker expression of MIAMI cells adhered onto LM-coated PAMs releasing BDNF was also studied in order to assess their potential therapeutic effect. We finally evaluated their behavior in an ex vivo organotypic model of HD. Therefore, the novelty of our paper lies in the combinatorial strategy using siREST silencing nanoparticles and cell transporting biodegradable polymeric PAMs releasing BDNF to achieve neuronally-committed MSCs releasing growth factors for a safe neuroprotective/neurorepair strategy for HD. 

## 2. Materials and Methods 

### 2.1. siRNA-LNCs

LNCs were formulated, as previously described [38] by mixing 20% *w*/*w* Labrafac^®^ WL 1349 (caprylic-capric acid triglycerides, Gatefossé S.A. Saint-Priest, France), 1.5% *w*/*w* Lipoid S75-3^®^ (Lecithin, Ludwigshafen, Germany), 17% *w*/*w* Kolliphor^®^ HS 15 (Polyethylene glycol-15-Hydroxystearate PEGHS BASF, Ludwigshafen, Germany), 1.8% *w*/*w* NaCl (Prolabo, Fontenay-sous-Bois, France) and 59.8% *w*/*w* water (obtained from a Milli-Q system, Millipore, Paris, France) together under magnetic stirring. Briefly, three temperature cycles between 60 and 95 °C were performed to obtain phase inversions of the emulsion. A subsequent rapid cooling and dilution with ice cold water (1:1.4) at the last phase inversion temperature led to blank LNC formation. For liposome preparation, a cationic lipid DOTAP (1,2-dioleyl-3-trimethylammoniumpropane) (Avanti^®^ Polar Lipids Inc., Alabaster, AL, USA), solubilized in chloroform, was mixed at a 1/1 molar ratio with the neutral lipid DOPE (1,2-dioleyl-sn-glycero-3-phosphoethanolamine) (Avanti^®^ Polar Lipids Inc.) to obtain a final concentration of 30 mM of cationic lipid. After chloroform vacuum evaporation, the lipid film was rehydrated and liposomes sonicated. A simple equivolume mix of liposomes and siRNA resulted in lipoplexes characterized by a charge ratio of 5 between the positive charge of lipids and the negative charge of nucleic acids. To obtain siRNA-LNCs, the water introduced at the last phase inversion temperature was replaced by lipoplexes, i.e., REST siRNA: (sense sequence: 5′-CAG-AGU-UCA-CAG-UGC-UAA-GAA -3′; Eurogentec, Seraing, Belgium) and control (scrambled) siRNA (sense sequence: 5′-UCUACGAGGCACGAGACUU-3′; Eurogentec) complexed with cationic liposomes in a defined charge ratio as described above. To avoid the possible denaturation of siRNA the addition of lipoplexes was performed at 40 °C.

### 2.2. Fluorescent siRNA-LNCs-DiD

To formulate fluorescent siRNA-LNCs, a solution of DiD (1,1′-dioctadecyl-3,3,3′,3′-tetramethylindodicarbocyanine perchlorate; em. = 644 nm; exc. = 665 nm) (Invitrogen, Cergy-Pontoise, France) solubilized in acetone at 25 mg/mL was prepared. 

For in vitro experiments, the DiD concentration was fixed at 200 µg/mL of LNC suspension or corresponding to 1.36 mg of DiD per grams of Labrafac^®^. The adequate volume of DiD solubilized in acetone was incorporated in Labrafac^®^ and acetone was evaporated at room temperature. The formulation process was unchanged, and formulation was stored at 4 °C, protected from light. For siRNA fluorescent LNCs, a fluorescent Alexa 488 siRNA (Eurogentec) was used.

### 2.3. BDNF-Releasing, Laminin (LM)-Coated PAMs

Synthesis and characterizations of PLGA-P188-PLGA polymer were performed using Synbio3 platform supported by GIS IBISA and ITMO Cancer. BDNF-releasing PAMs were prepared as previously described using a solid/oil/water emulsion solvent extraction-evaporation method [30]. Briefly, BDNF and human serum albumin were first nanoprecipitated separately and nanoprecipitated proteins were dispersed in the organic phase containing the polymer at a protein loading of 1 μg of protein and 5 μg of human serum albumin/mg of PAMs. The suspension was emulsified in a poly(vinyl alcohol) aqueous phase and after solvent extraction in an aqueous phase, the microspheres were filtered and freeze-dried. Blank microspheres, without protein, were prepared following a similar process. To obtain LM-covered PAMS (LM-PAMs), PLGA-P188-PLGA microspheres were coated with LM and poly-d-Lysine (PDL) as previously described [29]. Briefly, the coating solutions prepared in Dulbecco’s Phosphate-Buffered Saline (DPBS) were mixed under rotation with the microspheres at a final concentration of the coating molecules of 16 μg/mL of LM and 24 μg/mL of PDL (corresponding to a 40:60 ratio of LM:PDL). In vitro BDNF release from PAMs was performed as previously described by incubation of 5mg PAMs in citrate buffer and dosage by ELISA of collected fractions of the supernatant over time [30].

### 2.4. LNC and PAM Characterization

The size and Zeta potential of LNCs (*N* = 3) were measured by using the Dynamic Light Scattering (DLS) method using a Malvern Zetasizer^®^ apparatus (Nano Series ZS, Malvern Instruments S.A., Worcestershire, UK) after dilution at a ratio of 1:200 with deionized water. PAM’s size was measured with a Multisizer^®^ coulter counter (Beckman Coulter, Roissy France), zeta potential was measured by DLS [30]. The laminin surface was characterized by confocal microscopy (Leica TCS SP8, France) after LM immunostaining as previously described [30]. Lyophilized PAMs were incubated for 30 min at room temperature (RT) under 15 rpm stirring in DPBS containing 4% bovine serum albumin (BSA), 0.2% Tween 20 (DPBS BT). After washing, anti-LM mouse monoclonal antibody (Sigma-Aldrich, St-Louis, MO, USA, 100 μg/mL in DPBS) was added for 1.5 h under rotation at 37 °C. After washing, biotinylated anti-mouse IgG antibody (2.5 μg/mL in DPBS BT) was added for 1 h, at RT, washed and incubated with streptavidin–fluoroprobe 547 (1:1000 in DPBS) at RT, for 40 min. (*N* = 3, *n* = 3) 

### 2.5. MIAMI E/F Cells

MIAMI cells were isolated from human bone marrow (Lonza, donor #3515) and expanded on fibronectin (Sigma-Aldrich) coated flasks at 120 cells/cm^2^ in low oxygen tension (3% of O_2_ and 5% of CO_2_) in Dulbecco’s Modified Eagle Medium-low glucose (DMEM, Gibco, Life Technologies, Paisley, UK), supplemented with 3% of Foetal Bovine serum (FBS), 100 µM of ascorbic acid and a mixture of lipids, as previously described [20]. Using the same density of cells and culture condition, a 10 days treatment with an addition of 20 ng/mL of EGF and 20 ng/mL of FGF-2 (both from R&D systems, Lille, France) and 5 µg/mL of Heparin (Sigma-Aldrich) was used to enhance neuronal specification [23] and these cells were named MIAMI E/F cells. Every three days, half of the culture medium was replaced.

### 2.6. MIAMI E/F Cell Transfection

MIAMI E/F cells were seeded at 2000 to 3000 cells per cm^2^ in wells coated with LM (2 µg/cm^2^, Sigma-Aldrich). Experiments were performed in Opti-MEM^®^ media (Life technologies, France). SiRNA-LNCs were incubated with cells at 37 °C in a humidified atmosphere with 3% O_2_ and 5% CO_2_ for 4 h before serum addition. Cells were harvested after appropriate time and assayed for mRNA expression levels by RT-qPCR or protein expression level by immunofluorescence. 

### 2.7. LNC Cell Time Retention

MIAMI cells (2000 to 3000 cells per cm^2^) were seeded on glass slides coated with LM (2 µg/cm^2^, Sigma-Aldrich). SiRNA fluorescent LNCs were incubated with cells at 37 °C in a humidified atmosphere with 3% O_2_ and 5% CO_2_ for 4 h in Opti-MEM^®^ media before DPBS washing and paraformaldehyde fixation (4% of paraformaldehyde during 15 min at 4 °C) or FBS addition. At Day 0 and before fixation, 100nM LysoTracker Red (Molecular Probes, Eugene, OR, USA.) was added to the media. After washing, cells were visualized from Day 0 to Day 6 post-transfection using a fluorescence confocal multispectral imaging, FCSI (Leica TCS SP8). 

### 2.8. MIAMI E/F Cell Neuronal Differentiation

**Figure 1:**
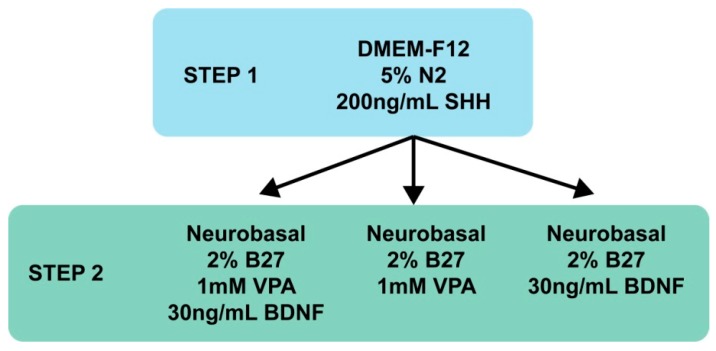
Media tested for the GABAergic differentiation of marrow-isolated adult multilineage inducible (MIAMI) cells.

MIAMI E/F transfected with siRNA-LNC cells were seeded (2000–3000 cells per cm^2^) on 175 cm^2^ cell culture flasks for Step 1 and on glass slides on 6 well plates for Step 2 coated with LM (2 µg/cm^2^, Sigma-Aldrich, St-Louis, MO, USA) and different conditions tested to obtain the best two-step GABAergic differentiation protocol (Figure 1). The first step was performed with DMEM/F12 (Glutamax, Gibco, Life Technologies, Paisley, UK) supplemented with 5% of N2 (1X) (both from Gibco, Life Technologies), and 200 ng/mL of sonic hedgehog (SHH, Peprotech, Rocky Hill, USA) for 14 days and 35 mL of media was used per flask. These cells are named MIAMI-SHH. Second step: Neurobasal media (Neurobasal, Gibco, Life Technologies) supplemented with or without 1 mM of Valproic acid (Sigma-Aldrich) and with or without 30 ng/mL of BDNF (Peprotech) for 14 days and 3 mL of media was used per well. Length and surface area were quantified using MetaVue software^®^. 6 pictures from each condition (24 in total) were taken with a 10× objective and used to determine total area and length. Only cells responding to the treatment (with neurite-like structures) were analyzed in this experiment.

### 2.9. Formation of PAMs-Cell Constructs

Cell adhesion studies were performed based on previous published protocols [22,24]. At the end of Step 1, MIAMI-SHH-siREST cells were detached and resuspended in DMEM-F12 supplemented with either 3% FBS (Lonza, Verviers, Belgium), 5% of N2 (1X) (Gibco, Life Technologies, Paisley, UK) or 2% of B27 (1X) (Gibco, Life Technologies). Lyophilized PAMs (0.5 mg) were resuspended in coated microcentrifuge tubes (Sigmacote, Sigma) containing DMEM-F12 (Gibco, Life Technologies), and mixed with 0.5 mL of cell suspension (2.5 × 10^5^ cells/0.50 mg PAMs). The mixture was then gently flushed and plated in 1.9 cm^2^ Costar ultra low adherence plate (#3473, Corning, Avon, France). Plates were incubated at 37 °C during 4 h for MIAMI E/F, to allow cell attachment on PAM surface (covered with FN). PAMs/cell aggregates were pelleted by centrifugation at 200 g for 2 min. Cell adhesion to PAM surface was assessed by microscopic observation and cells adhered to PAMs were quantified using the Cyquant cell proliferation assay (CyQuant Cell proliferation Assay kit, Invitrogen). Complexes were further studied using light and confocal microscopy and scanning electron microscopy. Samples were prepared for scanning electron microscopy analysis as previously described [24]. 

### 2.10. Preparation of Brain Organotypic Slices

Organotypic cultures were prepared as previously described by our team [39]. Briefly, Albinos wild-type Sprague–Dawley rats from the SCAHU (Service Commun d’Animalerie Hospitalo-Universitaire, N°49007002, Angers University, France) were used. Postnatal 9–11 (P9–11) days old Sprague–Dawley pups were used to generate the ex vivo model of HD. Animals were rapidly euthanized after anesthesia, by intraperitoneal injection of 80 mg/kg of ketamine (Clorketam 1000, Vetoquirol, Lure, France) and 10 mg/kg of xylazine (Rompum 2%, Bayer Health Care, Kiel, Germany). Brains were removed and rapidly dissected before being glued onto the chuck of a vibratome cooled with a bath of Gey’s balanced salt solution supplemented with 6.5 mg/L of glucose and antibiotics. 400 μm thick slices were cut using a vibratome (Motorized Advance Vibroslice MA752, Campdem instruments) in different configurations to obtain a progressive degeneration of the GABAergic medium spiny neurons (MSNs). Each hemisphere was mechanically separated to culture 8 organotypic brain slices total (4 from each hemispheres). Obtained slices were next transferred to 30 mm diameter semi-porous membrane inserts (Millicell-CM, Millipore, Guyancourt, France) within a 6-well plate, containing Neurobasal medium (Gibco, Life Technologies) supplemented with 6.5 mg/L of glucose, 1 mM of l-glutamine, 1x B27 supplements (Gibco, Life Technologies) and antibiotics. Slices were incubated at 37 °C and 5% CO_2_ up to 30 days and half of the medium was removed every 2–3 days. Each slice was cultured on a single membrane to increase their survival over time.

### 2.11. Injection of the Cells-PAMs Constructs into Organotypic Slides

Three days after organotypic slice preparation, the cells-PAMs were injected into the striatum using a 22-gauge Hamilton needle (Hamilton, Bonaduz, Switzerland) connected to a micromanipulator. The total injection volume consisted of 2 µL of culture medium containing approximately 75,000 cells alone or adhering to 0.1 mg of PAMs. The injections were done at an infusion rate of 0.5 µL/minute. The needle was left in place for 5 min before removal to avoid the cells being expelled from the organotypic slices.

### 2.12. Reverse Transcription and Real Time Quantitative PCR

Experiments were performed following the guidelines of the PACeM core facility ("Plate-forme d’Analyse Cellulaire et Moléculaire”, Angers, France). Total RNA of cells was extracted, purified using RNeasy Microkit (Qiagen, Courtaboeuf, France), treated with DNase (10 U DNase I/µg total RNA) and the concentration determined with a ND-2000 NanoDrop (Thermo Fisher Scientific, Wilmington, DE, USA). RNA integrity was verified on Experion RNA StdSens chip (Bio-Rad). First strand complementary DNA (cDNA) synthesis was performed with a SuperScriptTM II Reverse Transcriptase (Invitrogen), in combination with random hexamers, according to the manufacturer’s instructions. cDNAs were purified (Qiaquick PCR purification kit, Qiagen) and 3 ng of cDNA mixed with MaximaTM SYBR Green qPCR Master Mix (Fermentas) and primer mix (sense and antisense at 0.3 µM, Table 1 (Eurofins MWG Operon, Ebersberg, Germany) in a final volume of 10 µL. Amplification was carried out on a LightCycler 480 (Roche): denaturation step at 95 °C for 10 min and 40 cycles of 95 °C for 15 s, 60 °C for 30 s. Specificity of the primers was controlled. The GeNormTM freeware (http://medgen.ugent.be/-jvdesomp/genorm/) was used to choose GAPDH and ACTB, as the most stable housekeeping genes. The relative transcript quantity (Q) was determined by the delta Cq method: Q = E(Cq min in all the samples tested − Cq of the sample), where E is related to the primer efficiency (E = 2 if the primer efficiency = 100%). Relative quantities (Q) were normalized using the multiple normalization method (Vandesompele et al., 2002). Q normalized = Q/(geometric mean of the stable housekeeping genes Q). The 2(−ΔΔCt) method was retained, using a housekeeping gene and gene of interest (Livak and Schmittgen, 2001) tested on control sample and treated sample.

### 2.13. Immunocytofluorescence

After treatment, cells were fixed with 4% paraformaldehyde (PFA, Sigma, St Louis, MO, USA) in DPBS (Lonza, Verviers, Belgium) pH 7.4 during 15 min at 4 °C, washed and non-specific sites were blocked with DPBS, Triton 0.1% (DPBS-T, Triton X-100, Sigma), bovine serum albumin 4% (BSA, Fraction V, PAA Lab, Austria), normal goat serum 10% (NGS, Sigma) during 45 min at RT. A mouse anti-human β3-tubulin (2 µg/mL, clone SDL.3D10, Sigma), a mouse anti-human neurofilament medium (NFM, 1:50, clone NN18, Sigma-Aldrich), a monoclonal rabbit anti-human dopamine- and cAMP-regulated neuronal phosphoprotein (DARPP32, 0,6 µg/mL, clone EP721Y, Abcam, Paris, France), a mouse anti-glutamic acid decarboxylase-67 antibody (GAD67, 2 µg/mL, clone 1G10.2, Millipore SA), and a rabbit anti-GABA transporter 1 (GAT1 500 ng/mL Millipore SA) were used to characterize cell differentiation. Cells were incubated overnight with the primary antibody diluted into DPBS-T, BSA 4% at 4 °C. After washes, slices were incubated with the biotinylated mouse (2.5 µg/mL, Vector Laboratories, Burlingame, CA, USA) or rabbit secondary antibody (7.5 µg/mL, Vector Laboratories) for 1 hour at RT. Then slices were washed and incubated with Streptavidin Fluoroprobes 488 or 547H (Interchim, Montluçon, France) diluted 1:1000 or 1:500 respectively in DPBS for 1 h before mounting and observation using a fluorescence microscope. 

### 2.14. Immunofluorescence

Immunofluorescence of brain slices was performed as previously described [39]. Immunofluorescence was performed using antibodies against human mitochondria (hMito) (10 ng/mL, mitochondrial cytochrome C oxidase subunit II, Abcam), rabbit anti-human dopamine- and cAMP-regulated neuronal phosphoprotein DARPP32 (DARPP32, 0.6 µg/mL, clone EP721Y, Abcam) and a mouse anti-GAD67 (5 μg/mL, clone 1G10.2, Millipore SA). Isotypic controls were performed for each antibody. Free-floating slices were incubated in 1% DPBS-T (Sigma-Aldrich). After pre-blocking for 4 h with 4% BSA (fraction V, PAA Laboratories, Piscataway, NJ, USA), 10% NGS (Sigma-Aldrich) in DPBS-T, slices were incubated for 48 h at 4 °C with monoclonal antibodies against DARPP32, GAD67 or hMito with diluted in 4% BSA DPBS-T. After washing, the sections were incubated for 2 h slices with the biotinylated mouse (2.5 µg/mL, Vector Laboratorie,) or rabbit secondary antibody (7.5 µg/mL, Vector Laboratories) at room temperature. Then, slices were washed and incubated for 2 h with Streptavidin Fluoroprobes 488 or 547H (Interchim) diluted 1:500 or 1:1000 respectively in DPBS. Finally, the sections were washed mounted using fluorescent mounting medium (Dako, Carpinteria) and observed in a confocal microscopy.

### 2.15. MIAMI Cell Secretome Analysis

A Luminex^®^ Multiplex secretome assay was used to quantify cytokines and growth factors secreted by MIAMI cells under different conditions. 72 h before the experiment, media was completely removed and replaced by media without serum for MIAMI E/F or appropriate media for the other conditions. The media was collected and frozen at 72 h. The secretome of MIAMI cells was compared to the secretome of E/F pre-treated MIAMI cells cultured for 7–10 days. The secretome of E/F MIAMI cells was then analyzed after exposure to different conditions: after commitment into GABA-like progenitors (transfected by the si-REST and exposure during 14 days to SHH, and adhered on PAMS during 72H with Neurobasal media and 1 mM of Valproic acid. Eight human growth factors (Brain-derived neurotrophic factor (BDNF), beta polypeptide-Nerve growth factor (b-NGF), stem cell factor (SCF), Leukemia inhibitory factor (LIF), Hepatocyte growth factor (HGF), Placenta growth factor-1 (PlGF-1), Stromal cell-derived factor 1 alpha (SDF-1alpha), Vascular endothelial growth factor-A (VEGFa)) were quantified using 2 Luminex^®^ Multiplex assay panels: ProcartaPlex^®^ Human Chemokine Panel I 9 Plex (#EPX090-12187-901, ThermoFisher), ProcartaPlex^®^ Human Chemokine Panel 11Plex (#EPX110-12170-901, ThermoFisher). Samples were centrifuged at 4 °C for 10 min at 10,000 g and prepared as per the manufacturer’s recommendations using a Bio-Plex Pro wash station (Bio-Rad, Hercules, CA). No sample dilution was performed. Quantification of growth factors was performed on a Magpix apparatus (Bio-Rad) and analyzed with the Bio-Plex Manager Version 3.0 software (Bio-Rad). Appropriate media were used as control and to determine the background. Background subtraction was performed with appropriate media depending on the sample, *N* = 2, *n* = 2. N represents one independent experiment done at one moment using 375,000 MIAMI cells adhered to 0.5mg of PAMs for each condition tested, n is the number of samples for each condition. 

### 2.16. Data Analysis

Data are presented as the mean value of three independent experiments +/− standard deviation (SD) unless otherwise stated. Significant differences between samples were determined using an ANOVA test, followed by Dunnett’s multiple comparison tests, unless otherwise stated. Luminex data was analyzed with Kruskal–Wallis test followed by pair-wise comparison. Threshold *p*-value was set to 0.05 and significant differences was depicted with a “*”.

## 3. Results

### 3.1. MIAMI E/F Cell Transfection

**Figure 2:**
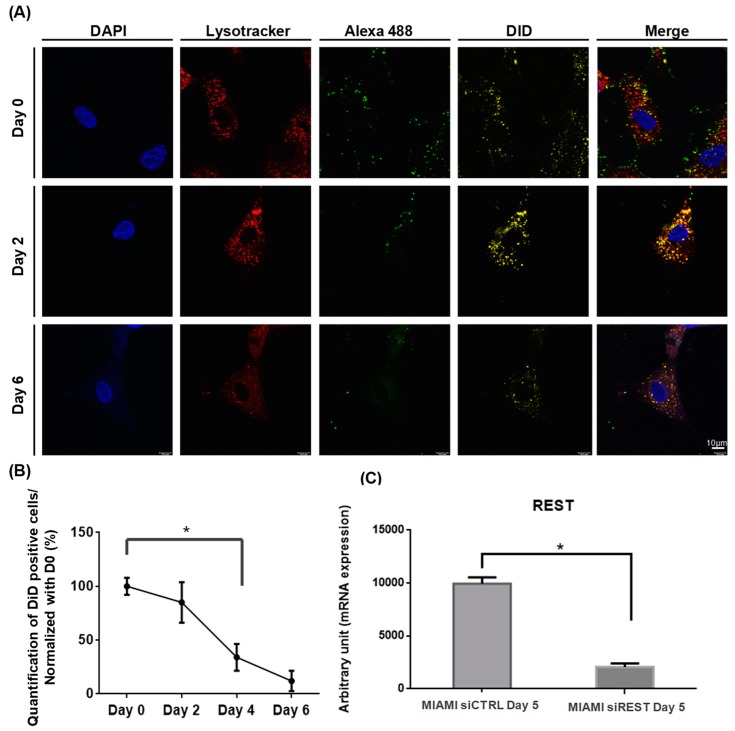
Cellular uptake and cell time retention of siRNA-LNC in MIAMI E/F cells. (**A**) After 4 h incubation, confocal microscopy was performed on MIAMI cells with siRNA-LNCs. Cells were fixed on glass slide and nucleus and lysosome staining were performed with DAPI (blue) and lysotracker (Red). Double fluorescent probes were used to follow siRNA and LNCs: lipophilic DiD (yellow) and Alexa488 siRNA (green). Scale bar represents 10µm. Analysis confirmed the internalization of siRNA-LNCs and its presence until Day 6. (**B**) DiD-positive cells representing the LNC-positive cells were counted using imageJ. 6 images per conditions in 10× objective were selected. (**C**) The expression of REST, measured by RT-qPCR, was decreased significatively (Fold decrease: 4.80 ± 1.80) at Day 5 after transfection compared to the control. *N* = 3. * Significantly different means at *p* < 0.05.

LNCs carrying REST siRNA of around 85 nm size and a positive surface charge of +7 mV were used for the incorporation of siREST into MIAMI E/F cells. To better understand the increase of neuronal markers in MSCs with siREST-LNCs [38], fluorescence confocal multispectral imaging was used to characterize LNC uptake and siRNA delivery on MIAMI E/F cells (Figure 2A). Triple-labeled LNCs, siRNA and lysosomes were generated, with respectively DiD, Alexa-488 and lysotracker to follow the distribution and localization of siRNA-LNCs over time. Four hours after the contact between LNCs and MIAMI E/F cells, referred to as Day 0, a heterogeneous distribution of LNCs within MIAMI E/F cells was observed (Figure 2A). LNCs (DiD-positive) co-localized with lysosome staining from Day 2 to Day 6 but the same co-localization of the siRNA (Alexa 488) was not observed confirming the results previously published by our team [40]. The number of fluorescent cells for the LNCs and siRNA decreased progressively from Day 0 until Day 9 (Figure 2B). Twelve percent of cells were still DiD-positive at Day 6 (Figure 2B). REST mRNA was quantified by RT-qPCR (Figure 2C) and down-regulated to 70.7% with the siREST at Day 5 when compared to the control cells (siCTRL), receiving a scrambled siRNA. 

### 3.2. MIAMI E/F Cells Commitment into GABA-Like Progenitors

**Figure 3:**
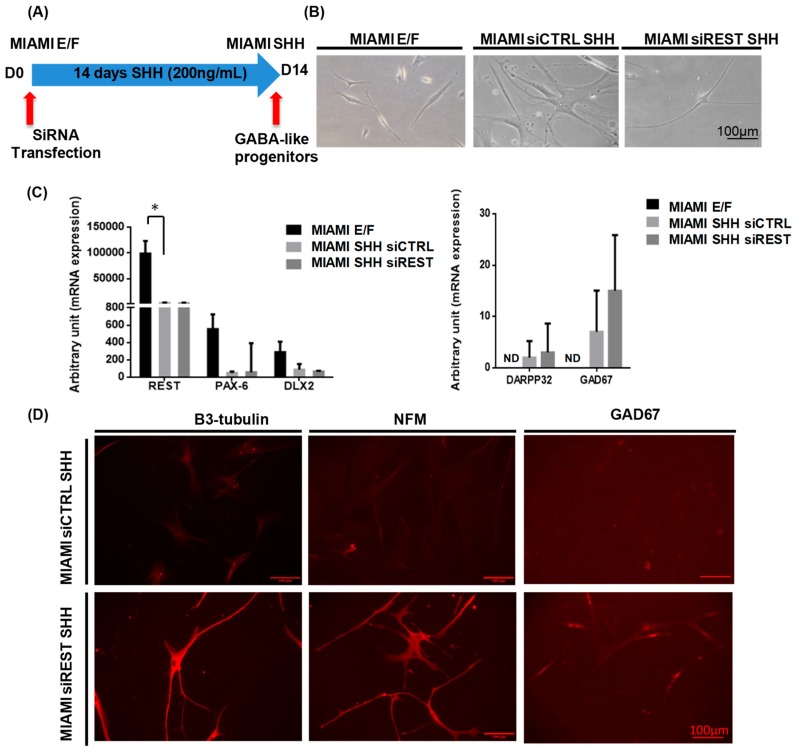
MIAMI E/F cell commitment into GABA-like progenitors. (**A**) For GABA-like progenitors commitment, a simple protocol has been designed using siREST and SHH. Transfection of MIAMI E/F was performed with 250 ng/mL of siCtrl and siREST-LNCs. (**B**) After the culture period of 14 days, MIAMI-SHH-siREST emitted long neuritis exhibiting a neuron-like morphology compared to the flat morphology of MIAMI-SHH-siCtrl. (**C**) The expression of neuronal commitment genes (REST, PAX-6, and Dlx2) and of genes of GABAergic-like neurons (DARPP32 and GAD67) was quantified at 14 days by RT-qPCR in MIAMI E/F, MIAMI-SHH-siREST and MIAMI-SHH-siCTRL cells (*N* = 3). (**D**) After immunofluorescence staining, the neuronal commitment of MIAMI-SHH-siREST cells was confirmed by the expression of β3-tubulin and NFM and a very slight expression of GAD67, all of which are not detected in MIAMI-SHH-siCTRL cells. ND: not detected. * Significantly different means at *P* < 0.05.

To ensure that the transplanted cells have a similar phenotype to the neurons in the striatum and thus warrant the safety of their transplantation in that area, the MIAMI E/F cells have been differentiated into GABA-like neurons. Cell density during the first steps is important and the confluence has to be maintained under 30% to induce cell differentiation. The MIAMI E/F cells were transfected with siREST or with a negative control, scrambled siRNA, followed by sonic hedgehog (SHH) treatment (Figure 3A). Some of the MIAMI E/F cells exhibited small neurite-like structures. Fourteen days after transfection, most of the MIAMI-SHH-siREST cells exhibited very long neurite-like structures. The majority of the MIAMI-SHH-siREST cells presented a total length of around 600 µm. In comparison MIAMI-SHH siCTRL cells showed a flat morphology (Figure 3B). REST expression, detected by RT-qPCR, was diminished over time during the treatment when compared to the MIAMI E/F cells. As expected at this time-point no difference in REST expression was observed between the cells transfected with siREST and siCtrl. Pax 6 and Dlx2, transcription factors detected in the ventral telencephalon, were expressed by MIAMI E/F cells and decreased slightly 14 days after the treatment (Figure 3C), The GABAergic marker, GAD67, was slightly increased following the treatment, at the mRNA and protein level (Figure 3C) and not detected in MIAMI E/F cells, as for DARPP32. No major differences were detected between the siREST and siCtrl conditions at this long-term time point, although there was a tendency in the MIAMI-SHH-siREST cells to express more GAD67 at the mRNA and protein level (Figure 3C,D). The early and late neuronal markers, β3-tubulin and NFM, respectively were expressed by the MIAMI-SHH-siREST cells and were only very slightly detected in MIAMI-SHH-siCtrl as shown by the immunofluorescence staining (Figure 3D). 

### 3.3. MIAMI E/F Cell Commitment into GABAergic-Like Neurons

**Figure 4:**
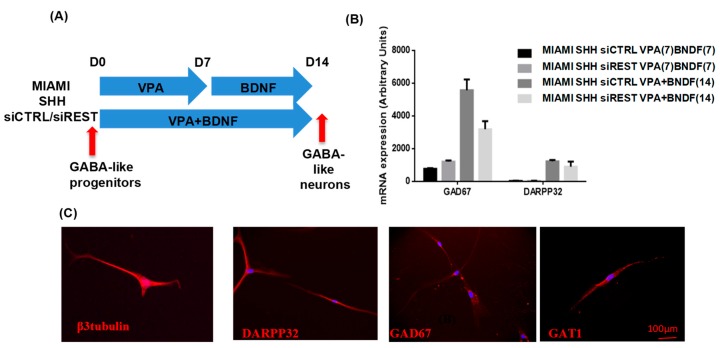
Differentiation and Characterization of GABA-like neurons. (**A**) Schematic procedure of GABAergic differentiation. For both protocols, the concentration of VPA was 10 mM and 30 ng/mL of BDNF). (**B**) The characterization of the differentiation was realized by RT-qPCR. The expression of genes DARPP32 and GAD67 was quantified at the end of the differentiation (*N* = 2, *n* = 1). (**C**) In vitro immunofluorescence against β3-tubulin, DARPP32, GAD67, GAT1 on GABA-like neurons was performed.

The GABA-like progenitors were exposed to Valproic acid (VPA) from Day 14 for seven days and BDNF from Day 21 for seven more days or during 15 days to VPA and BDNF in the same time, without SHH (Figure 4A) in order to obtain GABAergic-like neurons. At this step, no morphological change was observed between cells transfected with siCtrl or siREST, but the cells differentiated using VPA and BDNF in the same time exhibited longer neurite-like structure than the cells receiving the protocol using VPA and then BDNF (data not shown). In the same way, the expression of GABA markers detected by RT-qPCR (Figure 4B) is higher with the protocol using VPA together with BDNF compared to the sequential protocol (VPA then BDNF). At the end of the differentiation period, a low expression of Dlx2 and REST was detected by RT-qPCR (data not shown). Moreover, the inhibition of REST had no effect on the expression of GABAergic neuron markers with a tendency to be diminished for GAD67. The majority of the GABA-like neurons were positive β3-tubulin and some for DARPP32, GAD 67 and GAT1 after immunofluorescence, a feature of medium spiny neurons (Figure 4C).

### 3.4. Adherence of MIAMI E/F SHH-siREST on PAMs

**Figure 5:**
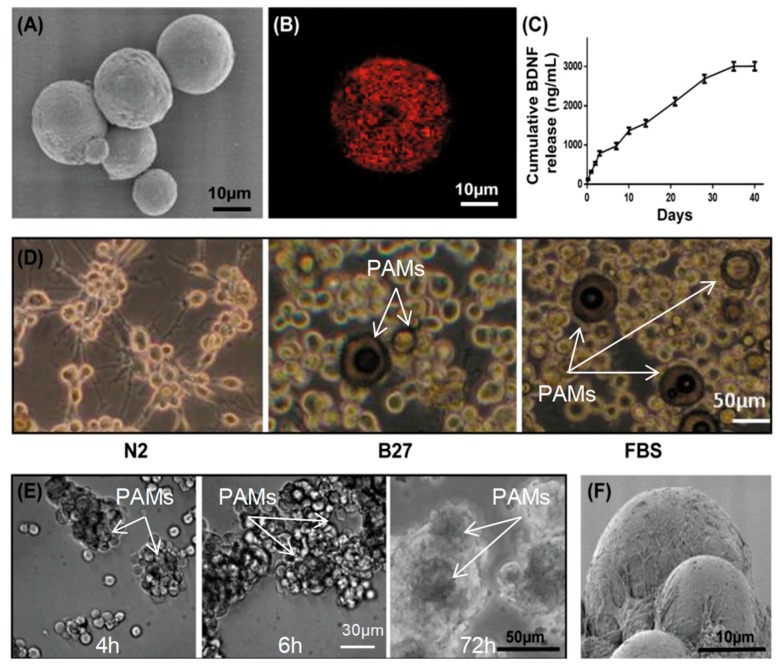
PAM characterization and adherence of GABA-like progenitors on PAMs. (**A**) PAMs observed using SEM and (**B**) laminin overlay onto PAMs observed by confocal microscopy after immunofluorescence. (**C**) Controlled release of BDNF from PAMs, measured using ELISA. (**D**) Different media were tested for the adher ence: 2% of B27, 15% of N2 and 3% of FBS. The last condition has been chosen for the rest of the experiments. (**E**) The cells were incubated with PAMs and pictures taken by brightfield microscopy at 4 h, 6 h and 72 h. (**F**) Observation of microspheres and cells-PAMs complexes by scanning electronic microscopy showing the cells adhering onto the PAMs. White arrows point at PAMs associated with cells.

The particle size of PAMs measured using a Multisizer Coulter Counter was around 30 µm, as also observed using SEM (Figure 5A). LM completely covered the whole surface of PAMs in small patches (Figure 5B) and a continuous release of BDNF was observed over time. Up to 3000 ng/mL of BDNF was progressively released during 40 days from 5 mg PAMs (Figure 5C). During the commitment and the differentiation, no serum and no antibiotics were used. In order to respect these conditions, different media for cell adherence were tested (Figure 5D). Unfortunately, a high proportion of cells were observed floating in the media or adhered to the plastic, particularly with N2 and also with B27 media (Figure 5D). On the contrary, with the 3% serum, the cells adhered nicely onto the PAMs (Figure 5D). Different times of adherence were tested, and it was observed that the adherence of the GABA-like progenitors increased over time; it was better after 6 h and 72 h in contact with PAMs compared to only 4 h (Figure 5E). At 4 h, a large quantity of cells was not combined to the PAMs, but cell/PAM aggregates could be observed from 6 h onwards. (Figure 5E). Since the cells completely surrounded the PAMs in Figure 5E, the PAMs could not directly be seen anymore. Using SEM, MIAMI E/F SHH-siREST cells were observed to adhere onto the PAMs with their lamellipodia surrounding the PAMs to form three-dimensional complexes (Figure 5F). The percentage of cells adhered onto PAMs’ surface at the end of the cell attachment protocol (72 h) was about 95.2 ± 2.7%, as measured using the CyQUANT DNA quantification method. 

### 3.5. Characterization of MIAMI-SHH-siREST on PAMs

**Figure 6:**
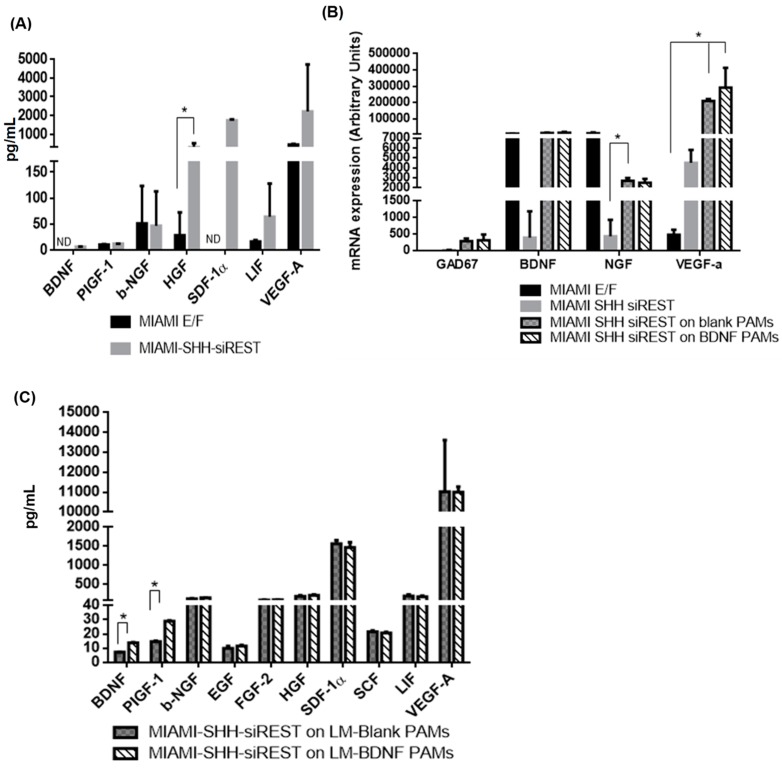
Characterization of the secretome profile of the GABA-like progenitors combined with PAMs. (**A**) Media collected from MIAMI E/F cells or MIAMI-SHH-SiREST cells were analyzed by the Luminex apparatus for the quantification of growth factors and cytokines (*N* = 2, *n* = 2). (**B**) The expression of GABAergic genes and GAD67 and growth factors (BDNF, NGF and VEGFa) was quantified by RT-qPCR 72H after adherence to the PAMs (*N* = 3). (**C**) Media collected 72 h after adherence between cells and PAMs were analyzed by the Luminex apparatus for the quantification of growth factors and cytokines (*N* = 2, *n* = 2) N represents one independent experiment with all the conditions, n the number of samples/condition. * Significantly different means at *P* < 0.05.

MIAMI E/F cells secreted a few growth factors, but interestingly SHH/siREST exposure affected the secretory profile of MIAMI E/F cells, which secreted more hepatocyte growth factor (HGF) and had a tendency to secrete more leukemia inhibitory factor (LIF) and vascular endothelial growth factor-a (VEGFa). They also secreted SDF-1α (Figure 6A, Table A1). As observed using RT-qPCR, the GABA-like progenitors expressed less BDNF and nerve growth factor (NGF), but after 72 h of adherence of these cells on the PAMs, the expression of BDNF, NGF and VEGFa increased (Figure 6B). The expression of GAD67 also tended to increase, while DARPP32 remained very low (23.00 ± 12.82 mRNA arbitrary units) At the protein level, the secretome analysis confirmed the release by the cells adhered onto PAMs of the above mentioned growth factors in the media as well as FGF2, EGF and stem cell factor (SCF). There was more placental growth factor (PIGF-1) and of course BDNF when the cells adhered onto PAMs releasing BDNF compared to blank PAMs (Figure 6C, Table A1). 

### 3.6. Behavior of PAMs and MIAMI-SHH-siREST in Huntington’s Disease Model

**Figure 7:**
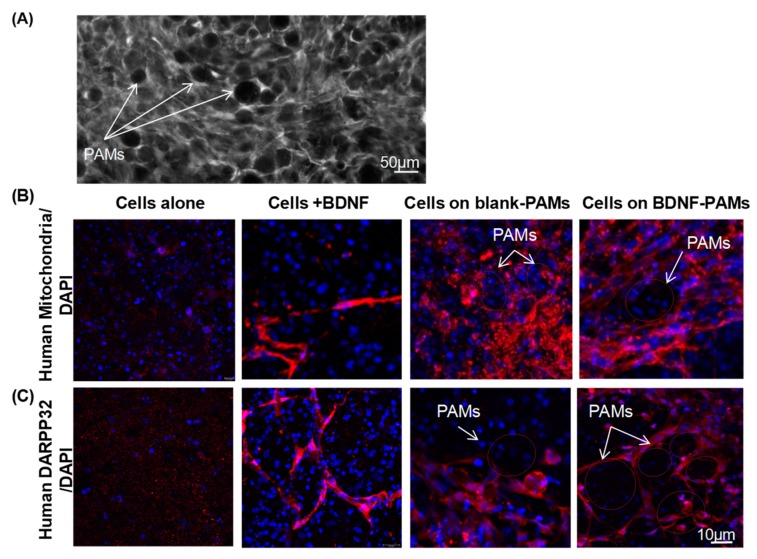
Behavior of MIAMI-SHH-siREST grafted into an ex vivo model of HD. (**A**) Black and white picture at lower magnification showing the PAMs with the transplanted cells within the tissue. All the cells are visualized by DAPI staining and the transplanted cells by immunohistofluorescence against human mitochondria. (**B**) Immunohistofluorescence against human mitochondria (in red) and (**C**) human DARPP32 (in red) for MIAMI cells 14 days after grafting, either alone, or exposed to BDNF for three days before grafting, or on LM-blank PAMs for three days before grafting or on LM-BDNF PAMs for three days before grafting. Red circles were suggested on the images to emphasize the localization of PAMs. DAPI staining (in blue) was used to visualize all the cells. Scale bar is 10 µm.

**Figure A1:**
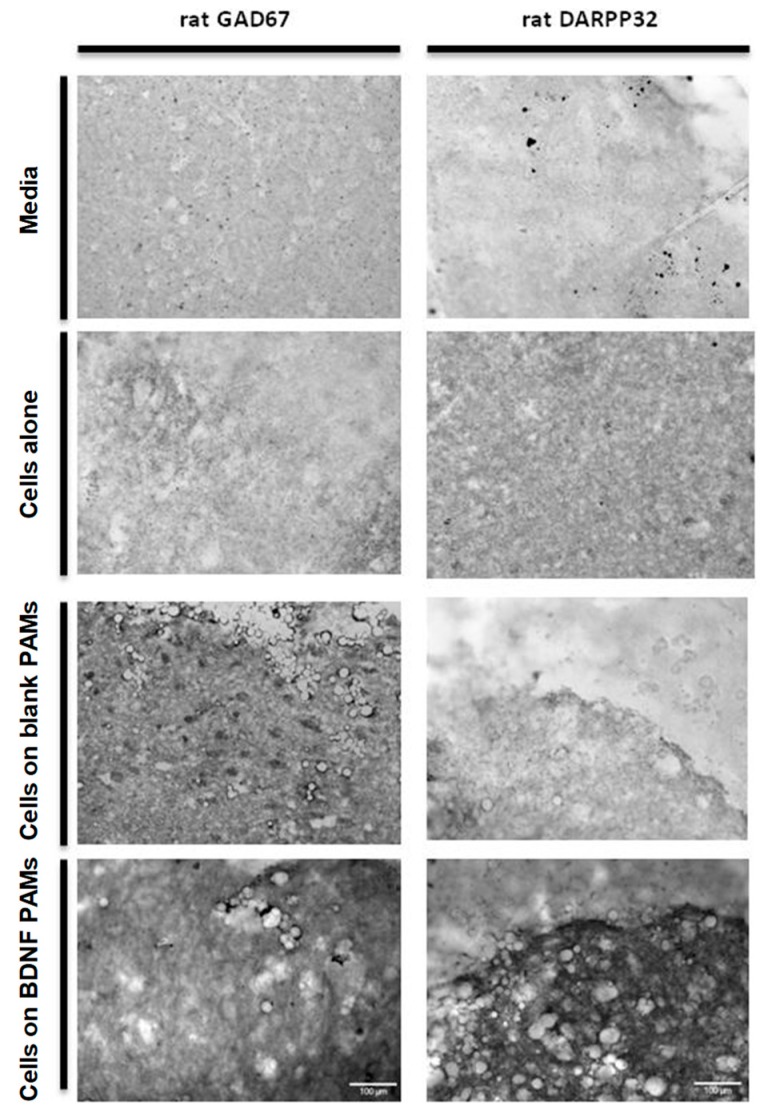
Neuroprotective effect of cells injection on the GABAergic degeneration. Immunohistochemistry against striatal GAD67-positive neurons and DARPP32-positive neurons at Day 25 after injection of media, cells alone, cells on blank PAMs and cells on BDNF PAMs in the ex vivo model of HD. Scale bar is 100µm (*N* = 2).

MIAMI-SHH-siREST alone, pre-treated with BDNF, or complexed to PAMs or BDNF-PAMS were grafted in the recently reported ex vivo HD model [39] at Day 5 when 30% of GABAergic striatal cell degeneration has occurred. Immunofluorescence against human mitochondria was used to visualize MIAMI-SHH-siREST cells in the rat brain (Figure 7A) and antibody against human DARPP32 was used to localize GABAergic-like neurons 15 days after grafting in the HD organotypic slices (Figure 7B). Immunofluorescence staining was faint with cells alone, suggesting that some cells died. PAMs delivering or not BDNF improved cell survival and BDNF administered to the cells before transplantation also seemed to slightly improve survival. Moreover, the cells receiving BDNF showed an elongated morphology with neurite-like structures (Figure 7A). Some of the transplanted cells expressed DARPP32 in all the conditions studied except for the cells transplanted alone (Figure 7B). GAD67 and DARPP32 immunostaining of the GABAergic rat striatal cells was more intense after transplantation of MIAMI-SHH-siREST cells on PAMs particularly when delivering BDNF (Appendix A Figure A1). 

## 4. Discussion

Mesenchymal stromal cell-based neuronal therapies can provide a limitless, easily accessible source of cells but survival and differentiation remain a drawback. Combinatorial strategies with nano- and microvectors designed to improve stem cell differentiation and engraftment are necessary. In this study, we used a combinatorial cell and drug delivery approach. We showed that a subpopulation of MSCs, the MIAMI cells, can differentiate towards the neuronal GABAergic lineage by an epigenetic RNA interfering approach inhibiting REST expression combined to GABAergic inducers. Moreover, GABA-committed MIAMI cells adhered onto PAMs maintained the GABAergic phenotype and secreted neural tissue repair factors. They could furthermore be transplanted while attached to PAMs delivering BDNF in an ex vivo model of HD, and were able to survive and maintain this GABAergic neuronal phenotype, particularly in response to BDNF. 

Our strategy to differentiate MIAMI cells into striatal-like neurons was developed based on the simplifying scheme that the normal course of neuronal differentiation may be separated into three successive steps: namely, (*i*) neural induction, (*ii*) regional commitment, and (*iii*) neuronal maturation. To obtain neural induction, we pre-treated MIAMI cells with EGF and FGF-2 as previously published by our group [23]. As expected, MIAMI E/F expressed Pax6 and slightly Dlx2, showing their progression towards a neural/neuronal phenotype. To induce a strong GABAergic commitment and further neuronal maturation, we chose to combine REST inhibition with adapted published protocols for pluripotent stem cells [41,42]. REST expression is progressively reduced during neuronal differentiation in neural stem cells [14] and its inhibition engaged MSCs into a neuronal pathway [15,38]. We chose to transiently inhibit REST with siREST carried by nanoparticles as previously performed [38]. Permanent down-regulation of REST with strategies such as ShREST would likely not be satisfactory since REST inhibition is only transient during normal brain development [43]. The siREST-LNCs inhibited REST expression for at least 5 days, probably due to their long retention time within the cells. After this transient REST inhibition together with SHH treatment followed by the combination of VPA and BDNF, we first obtained GABA-like progenitors and then immature neurons expressing striatal markers such as GAD67, DARPP32 and GAT1. The main difference observed after REST inhibition was a clear neuronal commitment of the GABAergic-like progenitors, which expressed the neuronal markers B3-tubulin and NFM. To our knowledge, our strategy is the first to describe a direct non-epigenetic transient modulation of REST with nanoparticles to direct MSCs toward a GABAergic lineage. Other approaches used to inhibit REST are burdened by intrinsic limitations such as the HIV origin of the product and host genome integrations of lentivirus or oligodeoxynucleotides, which may lead to unwanted side effects.

MIAMI cells committed or not towards a neuronal phenotype easily adhere onto PLGA PAMs with a biomimetic surface composed of extracellular matrix molecules in the presence of low serum concentrations [26,29,30]. Different serum free media were evaluated for the formation of PAM/cell complexes to avoid washing steps before implantation to dispose of the undesired serum. Moreover, in this way, the implantation procedure is simplified, and the cells implanted in their own conditioned media already containing some BDNF secreted by the cells and delivered from the PAMS. However, the GABA-like progenitor cells only adhered to PLGA-P188-PLGA PAMs with a LM biomimetic surface in the presence of 3% serum. In this study, we observed that MIAMI E/F derived GABA-like progenitors express more GAD67, a constitutive striatal marker, when adhered onto PAMs compared to the cells in a 2D culture. The laminin-covered PAMs stimulate this differentiation as it has been shown that laminin stimulates neuronal differentiation of MSCs [44] and of MIAMI cells [8]. Nevertheless, the 3D condition should also contribute as our previous results showed that PAMs offering a 3D biomimetic surface stimulate MIAMI cells neuronal differentiation [30]. In a similar manner neural stem/progenitor cells also more efficiently differentiated to neurons in a collagen/hyaluronan 3D matrix compared to a 2D culture condition [45].

MSCs have recently emerged as a promising cell population to protect degenerating neuronal cells and increase the function of the remaining cells in various neuronal disorders. In HD preclinical models, the functional recovery observed after MSC transplantation could be explained by the secretion of neurotrophic factors including BDNF, CNTF, NGF, insulin-like growth factor 1 and EGF [10,11]. Within this line, previous studies have shown that MIAMI cells, which are a homogeneous population of cells with an unique genetic profile, express several pluripotency markers (Oct4, Sox2, Nanog, SSEA4) and other proteins also expressed by embryonic stem cells, release more tissue repair factors than MSCs or embryonic stem cells and protect the neurovasculature [45]. Compared to naïve MIAMI cells, MIAMI cells pre-treated with E/F secreted more tissue repair factors such as VEGFA, NGF, LIF and HGF [30], and we observed that SHH/siREST treatment further increased the expression of HGF, but also of BDNF and SDF-1α. SDF-1α is an indispensable chemoattractant for neuron migration in different brain regions (CXCR4 regulates interneuron migration in the developing neocortex.). Moreover, it has been demonstrated that SDF-1 coexists in GABA-containing vesicles in the terminals of basket cells, regulates the strength of GABAergic input to nestin^+^-type 2 neural progenitors in hippocampal dentate gyrus, and plays a crucial role in adult neurogenesis [46], while laminin also enhances the chemotactic activity of SDF-1 in the thymus [47].

We previously demonstrated that PAMs delivering neurotrophin-3 can improve MIAMI cells survival, thereby preserving neural function in a Parkinson’s disease animal model [8,22]. PAMs encapsulating BDNF and composed of a triblock copolymer of PLGA-P188-PLGA were used in this study as we previously showed that it allowed for a complete release of functionally active growth factors from the microspheres [28,30]. Exposure of MIAMI E/F cells to SHH/siREST and adhesion to PAMs had a drastic effect on the expression of NGF and VEGF at the mRNA level, and we also observed an augmentation at the protein level of BDNF and PIGF-1 in the media when cells were attached to BDNF-loaded PAMs compared to blank PAMs. The increase in BDNF in the media was likely due to the release of BDNF from PAMs since no difference was observed at the mRNA level in MIAMI SHH/siREST attached to PAMs with or without encapsulated BDNF. VEGF and NGF are known to increase neurogenesis [48], while VEGF, NGF, and PIGF-1 also all share in common the possibility of being of benefit to enhance reparative angiogenesis [49,50]. In this regard, we demonstrated in another study that VEGF secreted by MIAMI cells can improve vascularization maintenance after grafting in an ex vivo model of Parkinson’s disease [22]. These findings thus support the use of PAMs to enhance the benefit of MIAMI SHH/siREST in the context of HD. In this study, we tested this innovative strategy combining mesenchymal stromal cells and biomaterials in an ex vivo model of HD [39]. The benefit of this model is that it preserves cell graft-host tissue crosstalk, while maintaining the cytoarchitecture of the original tissue. We here demonstrated the screening capacity of this HD model for new therapeutic strategies. After grafting, GABA-like progenitors alone did not survive while if associated to PAMs the 3D support seemed to stimulate the survival of the cells after transplantation as already demonstrated by our group [22,23,24]. BDNF pre-treatment also stimulated cell survival while the combination of the BNDF delivery and the 3D polymeric support, provided by the PAMs, seemed to further enhance this survival. Moreover, these GABA-like progenitors submitted to BDNF, known to induce neuronal survival and differentiation [30,31,32] showed an elongated morphology with neurite-like structures suggesting the maintenance of their neuronal phenotype. In this regard, they also expressed DARPP32, a GABAergic marker. Finally, the combination of cells and PAMs delivering BDNF seemed to delay the degeneration of GABAergic neurons in the HD model, thereby demonstrating the potential benefits of this strategy for the treatment of HD. Further studies in a transgenic mouse model of HD are however needed to obtain confirmation that this combinatorial therapeutic strategy leads to neurorepair and provides a functional benefit.

## 5. Conclusions

We here demonstrated the therapeutic potential of this novel combinatorial strategy using LNC-delivered siREST and neuronally-committed MSCs transported by laminin-covered microcarriers delivering BDNF. We showed the capacity of MIAMI E/F cells to differentiate into GABAergic-like neurons by using REST inhibition and the appropriate media cues in vitro. GABA-like progenitors adhered to PAMs increased their mRNA expression of NGF/VEGF-a as well as their secretion of PIGF-1, three chemokines known for their potential to enhance reparative angiogenesis. Together, our results indicate that MIAMI cell/PAMs complexes survive after transplantation in an ex vivo HD model, maintain a GABAergic-like phenotype and may alleviate cell damage in the context of HD through chemokine secretion.

## Figures and Tables

**Table 1:** Primer sequences used for RT-qPCR.

Gene	Full name	NM number	Sequences
ACTB	Actin	NM_001101.3	F: CCAGATCATGTTTGAGACCT
			R: GGCATACCCCTCGTAGAT
BDNF	Brain-derived neurotrophic factor	NM_001143816	F: CAAACATCCGAGGACAAGG
			R: TACTGAGCATCACCCTGG
DARPP32	Dopamine- and cAMP-regulated phosphoprotein	NM_181505	F: GAGAGCCTCAGGAGAGGG
			R: CTCATTCAAATTGCTGATAGACTGC
Dlx2	Distal-less homeobox 2	NM_004405	F: GACCTTGAGCCTGAAATTCG
			R: ACCTGAGTCTGGGTGAGG
GAD67	Glutamic Acid Decarboxylase 67	NM_000817	F: GGTGGCTCCAAAAATCAAAGC
			R: CAATGTCAGACTGGGTAGCG
GAPDH	glyceraldehyde-3-phosphate dehydrogenase	NM_001289745.1	F: CAAAAGGGTCATCATCTCTGC
			R: AGTTGTCATGGATGACCTTGG
GDNF	Glial cell line-derived neurotrophic factor	NM_011675.2	Qiagen, ref #QT00001589
Pax6	Paired box 6	NM_000280	F: TTTCAGCACCAGTGTCTACC
			R: TAGGTATCATAACTCCGCCC
NGF	Nerve growth factor	NM_002506	Qiagen, ref #QT00043330
REST	RE1-silencing transcription factor	NM_001193508.1	F: ACTCATACAGGAGAACGCC
			R: GTGAACCTGTCTTGCATGG
VEGFA	Vascular endothelial growth factor A	NM_001204384	F: CAGCGCAGCTACTGCCATCCA
			R: CAGTGGGCACACACTCCAGGC
ACTB	Actin	NM_001101.3	F: CCAGATCATGTTTGAGACCT
			R: GGCATACCCCTCGTAGAT

## Appendix A

***Immunohistochemistry.*** A mouse anti-GAD67 (5 µg/mL, clone 1G10.2, Millipore SA, Guyancourt, France) and a mouse anti-DARPP32 (0.25 µg/mL, clone 15, BD Bioscience, Le Pont de Claix, France) antibodies were used to observe striatal-GP GABAergic neurons. Isotypic antibodies were used to control background staining. After 2 h of saturation in DPBS, 4% BSA, 1% triton and 10% NGS, slices were incubated 48 h with the primary antibody diluted in DPBS, 4% BSA and 1% triton at 4 °C. After washes, slices were incubated with the biotinylated anti-mouse secondary antibody (2.5 µg/mL Vector Laboratories, Burlingame, CA, USA). Then slices were washed, and quenching of peroxidase was performed with 0.3% H_2_O_2_ (Sigma, Saint Quentin Fallavier, France) in DPBS, at room temperature for 20 min. After DPBS washes, slices were incubated with Vectastain ABC reagent (Vector Laboratories, Eurobio, Les Ulis, France) in DPBS at room temperature for 2 h. Sections were then washed and revealed with 0.03% H_2_O_2_, 0.4 mg/mL diaminobenzidine (DAB, Sigma, Saint Quentin Fallavier, France) in DPBS, 2.5% nickel chloride (Sigma, Saint Quentin Fallavier, France) and dehydrated before mounting.

**Table A1:** Raw data of the secrotome analysis. Media collected from MIAMI E/F cells, MIAMI-SHH-SiREST cells or 72 h after adherence between cells and PAMs were analyzed by the Luminex apparatus for the quantification of growth factors and cytokines (*N* = 2, *n* = 2).

Heading	BDNF	PIGF-1	b-NGF	HGF	SDF-1	LIF	VEGF-A
MIAMI E/F media (Control)	4.55 ± 0.294	14.36 ± 0.20	0.00	124.93 ± 1.52	481.95 ± 3.27	49.35 ± 3.43	34.21 ± 0.676
Supernatant MIAMI E/F	1.85 ± 0.139	24.38 ± 1.25	51.07 ± 72.22	152.87 ± 42.87	385.73 ± 52.87	65.80 ± 2.54	482.53 ± 32.54
Step 1 Media (Control)	0.00	0.00	0.00	0.00	13.61 ±0.65	0.00	0.00
supernatant MIAMI E/F SHH siREST	5.95 ± 0.59	11.70 ± 0.26	46.68 ± 66.05	304.12 ± 216.21	1746.64 ± 63.87	63.78 ± 64.24	2205.48 ± 505.54
Step 2 Media without BDNF (Control)	0.00	0.00	0.00	0.00	14.76 ± 0.01	0.00	0.00
supernatant MIAMI E/F SHH siREST on LM-blanks PAMS	7.21 ± 0.19	14.73 ± 0.46	128.04 ± 12.21	192.08 ± 25.37	1567.67 ± 92.07	195 ± 39.81	11,028.92 ± 2576.19
supernatant MIAMI E/F SHH siREST on LM-BDNF PAMS	13.77 ± 0.35	29.02 ± 0.41	148.30 ± 6.18	219.43 ± 15.76	1467 ± 138.48	180.11 ± 20.07	11,003.58 ± 1189.29
